# Supplementary material for: Heterogeneously integrated ITO plasmonic Mach–Zehnder interferometric modulator on SOI
Source: Sci Rep. 2021 Jan 14;11:1287. doi: 10.1038/s41598-020-80381-3 (PMC7809469; doi:10.1038/s41598-020-80381-3)
Supplement: Supplementary file 1 — Supplementary Information [file 41598_2020_80381_MOESM1_ESM.pdf]

Supplementary Information: Heterogeneously Integrated ITO Plasmonic Mach-Zehnder Interferometric Modulator on SOI

Rubab Amin[1], Rishi Maiti[1], Yaliang Gui[1], Can Suer[1], Mario Miscuglio[1], Elham Heidari[2], Jacob B. Khurgin[3], Ray T. Chen[2], Hamed Dalir[4], and Volker J Sorger[1,*]

[1]*Department of Electrical and Computer Engineering, George Washington University, Washington, DC 20052, USA*
[2]*Microelectronics Research Center, Electrical and Computer Engineering Department, University of Texas at Austin, Austin, Texas 78758, USA*
[3]*Department of Electrical and Computer Engineering, Johns Hopkins University, Baltimore, Maryland 21218, USA*
[4]*Omega Optics, Inc. 8500 Shoal Creek Blvd., Bldg. 4, Suite 200, Austin, Texas 78757, USA*
*Corresponding author: sorger@gwu.edu

1. Fabrication Process Flow

The passive Si structures e.g. Mach-Zehnder interferometer with waveguides and grating couplers were taped out. A 5 nm thin film of Al_2O_3 was deposited as passivation layer for the passive structures and to aid the grating coupler environmental coupling efficiency. Pattern transfers for the active region were performed via e-beam lithography (EBL). A 10 nm thin film of ITO is deposited using an ion beam deposition (IBD) process. The ITO metal contact is then set forth by EBL patterning, e-beam evaporation and necessary liftoff processes. We used Au metal contacts with a thin Ti adhesion layer, corresponding thicknesses of 47 nm and 3 nm respectively. Since the modulation efficiency (ER/V_{pp}) is improved with respect to electrostatics, we opted to use a relatively high-k dielectric, a 20 nm oxide layer of Al_2O_3 is grown on the passive structure using atomic layer deposition (ALD) to aid capacitive gating schemes. Since the ALD process does not allow pattered deposition due to chamber contamination concerns, the gate oxide film did cover the ITO contact also. Subsequent patterning on top of the ITO contacts laid out windows for electrical probing. An wet etch step (MF319) was required for opening the oxide on top of the ITO

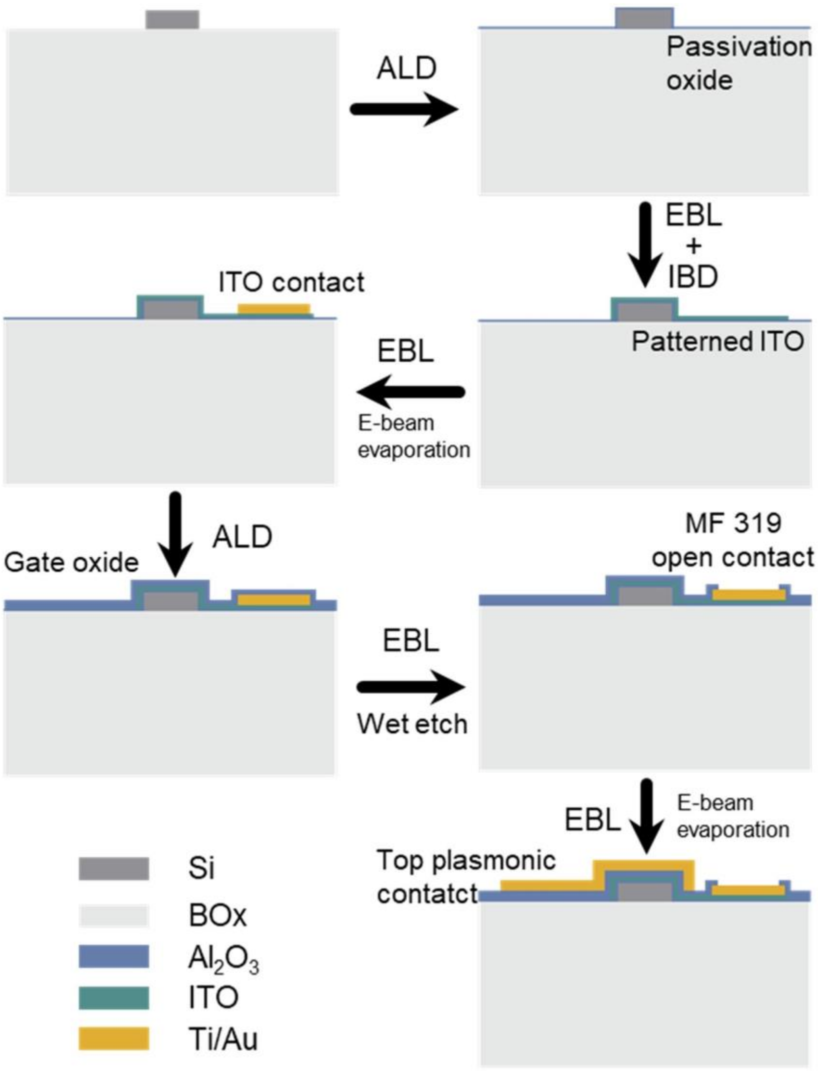

Figure S1: Fabrication process flow. The entire process flow from the passive waveguide structures to the final active device formation is shown here with the necessary nanofabrication processes outlined step by step.

metal contact in order to efficiently probe the device electrically. Finally, patterns for the top plasmonic metal contact were carried out via another EBL step followed by e-beam evaporation and liftoff steps. Contacts and the plasmonic top layer are formed by depositing 50 nm of Au using electron beam evaporation process. An adhesion layer of 3 nm of Ti is used in the process. The chip was finally spin coated with PMMA based resists once more as the PMMA refractive index aids in coupling efficiency of the light to/from the grating couplers compared to just air. One final

EBL step was undertaken to create windows on the PMMA layer so that the electrical probes would not get contaminated with PMMA resists while measuring.

2. Fabricated Device Scaling

Active phase shifters with three different device lengths are fabricated sweeping from the sub-λ regime to λ-scale devices comprising of 1.4 μm, 2.5 μm and 3.5 μm long phase shifters.

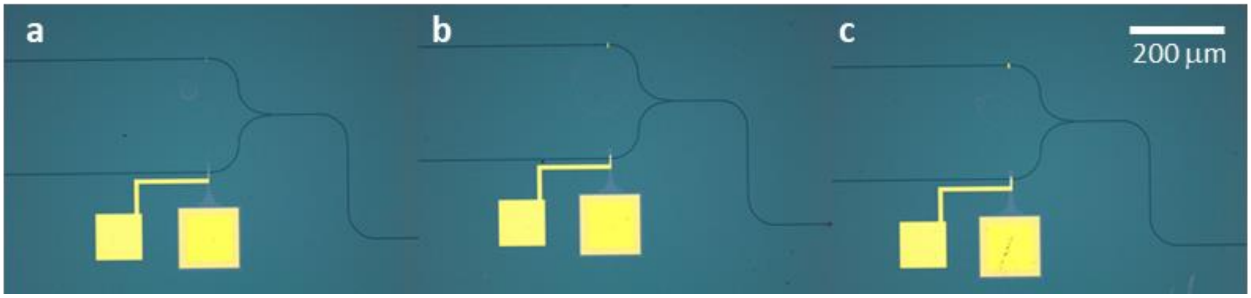

Figure S2: Fabricated devices scaling. Active device lengths are swept in length of **a.** 1.4 μm; **b.** 2.5 μm; and **c.** 3.5 μm. The other arm of the Mach-Zehnder structure is also loss-balanced by passive plasmonic metallic strips accordingly.

3. Ellipsometry Fitting Detail

In the following section we provide an accurate description of the method used for fitting the ellipsometry data using a model (Cauchy, Drude, Lorentz and Cody-Lorentz) and suitable algorithm and figure of merit, which allowed to determine fundamental parameters of the investigated film, optimizing the goodness of the fit.

A. Cauchy model

In our work, we use Cauchy model to fit the transparent region and find out the thickness of the thin film according to the following:

$$n(\lambda) = A + \frac{B}{\lambda^2} + \frac{C}{\lambda^4} \quad \text{(S1)}$$

where A, B, C are adjusted to fit the refractive index for this region. Since Cauchy model is not constrained by Kramer-Kronig relation, the un-physical shape should be neglected. The advantage

of Cauchy model is that it has only 2-3 free parameters are needed to achieve the fitting. But the limitation is that it can only be used in transparent region.

B. B-spline

We used the B-spline to expand the fitting wavelength. This kind of fitting was unrelated to the physics involved and exploited a nth degree polynomial:

$$P_m(x) = a_m x^m + a_{m-1} x^{(m-1)} + \cdots + a_1 x + a_0 \tag{S2}$$

B-spline is designed to best match the known shape of optical constant in whole range, while Cauchy model can only describe the transparent region. Comparing with GenOsc model, B-spline can describe more optical function shapes.

C. GenOsc model

For addressing peaks in absorption at resonant frequencies where the material is most likely to absorb the incoming light of that wavelength, we use Genosc model. In GenOsc model, oscillator equations are used to describe resonant absorption. The permittivity function can be described as:

$$\Im\big(\varepsilon(f)\big) = \Im(\varepsilon_{\text{Drude}}) + \Im(\varepsilon_{\text{Lorentz}}) + \Im(\varepsilon_{\text{Tauc-Lorentz}}) \tag{S3}$$

which consists of the summation of the imaginary part of a Drude oscillator function, matching the lower frequency, the Lorentz oscillator function and a Tauc Lorentz oscillator function matching the higher frequency peak.

I. Drude oscillator

Drude oscillator is based on the classical equations of motion of an electron in an optical electron in an optical electric field, and fives the simplest theory of the optical constants. The Drude model portion [1] of complex permittivity is:

$$\varepsilon_{\text{Drude,Ellipsometry}} = \frac{\hbar}{\varepsilon_0 \rho(\tau E^2 - i\hbar E)} \tag{S4}$$

where $\hbar$ is the reduced Planck's constant, ε_0 is the free space permittivity and E is light energy.

II. Lorentz and Tauc-Lorentz oscillator [2]

Lorentz oscillators are characterized by board absorption and it is suitable for describing metals. They are used to model excess absorption near the bulk plasma frequency besides Drude model. The portion of Lorentz oscillator [2] can be described as:

$$\varepsilon_{\text{Lorentz,Ellipsometry}} = A_1 \frac{f_{1,r} f_{1,c}}{f_{1,c}^2 - f^2 - i f_{1,r} f)} \tag{S5}$$

Both Tauc-Lorentz and Cody-Lorentz can be used to describe UV region of ITO thin film. In this work, we use Tauc-Lorentz. The portion of Tauc Lorentz oscillator can be described as:

$$\varepsilon_{Tauc-Lorentz,Im} = \begin{cases} \frac{A_2}{f} \frac{f_{2,c} f_{2,r} \left(f_g - f\right)^2}{\left(f_{2,c}^2 - f^2\right)^2 + f_{2,r}^2 f}, & f \geq f_g \\ 0, \; f < f_g \end{cases} \tag{S6}$$

where A_n is the unitless amplitude of the oscillator, $f_{n,r}$ and $f_{n,c}$ represent the broadening and central frequency of the oscillator, respectively, for the Lorentz (n = 1) and Tauc−Lorentz (n = 2) models, and f_g represents the band-edge frequency of the Tauc−Lorentz oscillator.

4. DC Measurement Setup

The electro-optic measurement setup used for our obtained results is depicted in Fig. S2. For single wavelength operation, λ =1550 nm was chosen for telecom relevance. Input light from the tunable laser (Agilent 81950A) was coupled into the device with optical fibers and with the help of transverse magnetic (TM) grating couplers. Similarly output gfrom the device was coupled to a fiber using same gratings and collected at the photodetector (Agilent 81624B). The applied bias for modulation was provided using a DC source (Keithley 2601B) and DC probes (Signatone). Similar setup was utilized in measuring the spectral response as well switching the tunable laser with a broadband erbium doped fiber amplifier (EDFA) source (AEDFA-PA30-8-FA) and collecting the output with the help of an optical spectrum analyzer (OSA 202).

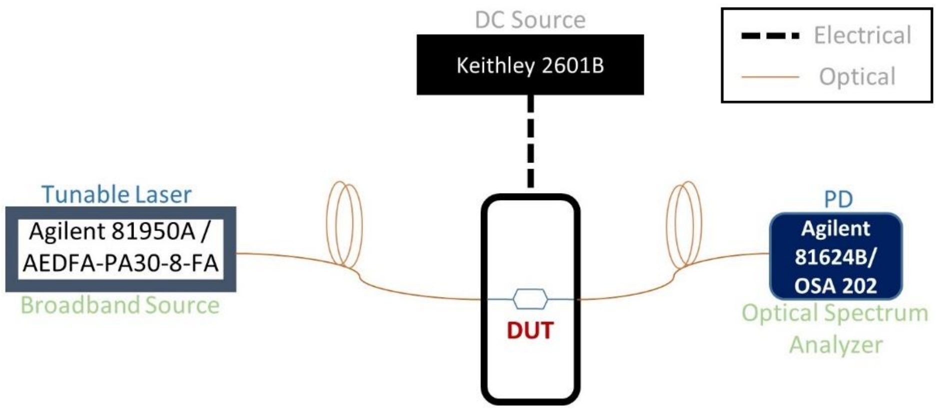

Figure S3: The electro-optic DC measurement setup. Telecom relevant input light of 1550 nm from the tunable laser is coupled to the device under test (DUT) via optical fibers and transverse magnetic (TM) grating couplers and output is similarly coupled to another fiber with TM grating couplers to the photodetector (PD) for single wavelength operation. The broadband spectrum response is measured in a similar manner using the erbium doped fiber amplifier (EDFA) as a broadband source and collecting the output with an optical spectrum analyzer (OSA). The bias voltage was provided using the DC source.

The measurement setup used a probe station and optical microscope for probing the device electrically and aligning the optical input and output fibers with corresponding grating structures. This measurement setup was used extensively for reliable measurement of the results and repeatability.

5. Wet Etch of Al_2O_3

The use of atomic layer deposition (ALD) to grow 20 nm of Al_2O_3 gate oxide in our process essentially imposes an etch step on top of the Si contact pad for electrical probing as the ALD tool does not allow photoresist patterning due to the contamination of the chamber. We employed an wet etch method using MF319 containing tetramethylammonium hydroxide (TMAH) which reacts with the Al and can etch the oxide thereof, but this is a rather slow process. To find the optimal etching rate of Al_2O_3, a sample was prepared depositing 3 nm of Ti and 47 nm of Au using e-beam evaporation and subsequently 20 nm of Al_2O_3 was grown using ALD at 100°C emulating the actual device contacts.

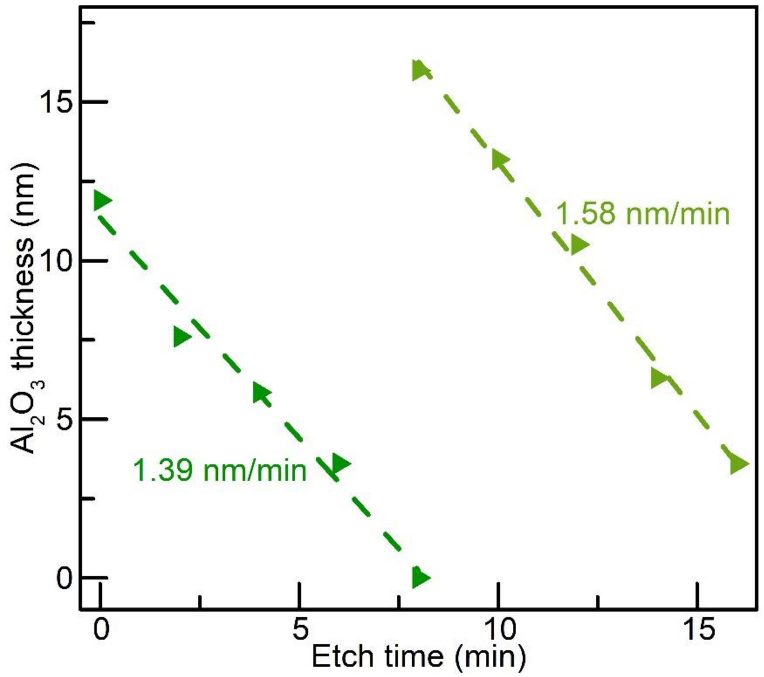

Figure S4: Al_2O_3 etch rate in the wet etch method using MF319 solution. Etching on top of contacts allowed us to neglect the etch selectivity as the contacts underneath act as hard masks. This etching process is rather slow than Al etching using similar solution.

This sample was placed in an MF319 solution for varying lengths of time. After 2 minute intervals, the sample was taken out, cleaned with de-ionized (DI) water and isopropyl alcohol (IPA) to prevent further etching or undercuts, then measured using a general-purpose film thickness measurement instrument, Filmetrics F20-UV. After each interval measured, we expected to see a decrease in Al_2O_3 thickness and we found the etch rate roughly around 1.49±0.13 nm/minute.

6. ITO Material Metrology

To determine the contact resistance, sheet resistance and resistivity of ITO on glass, transmission line measurements (TLM) were conducted. First, a 10nm thin film of ITO was deposited on a glass substrate using the IBD tool keeping all process parameters fixed matching the exact process used on the actual device. Subsequently, several 100 μm × 100 μm contact pads

were patterned linearly spaced 100 μm apart on it using the electron beam lithography (EBL) tool (Raith VOYAGER) emulating contact pads on the device. Two probes in the TLM setup were

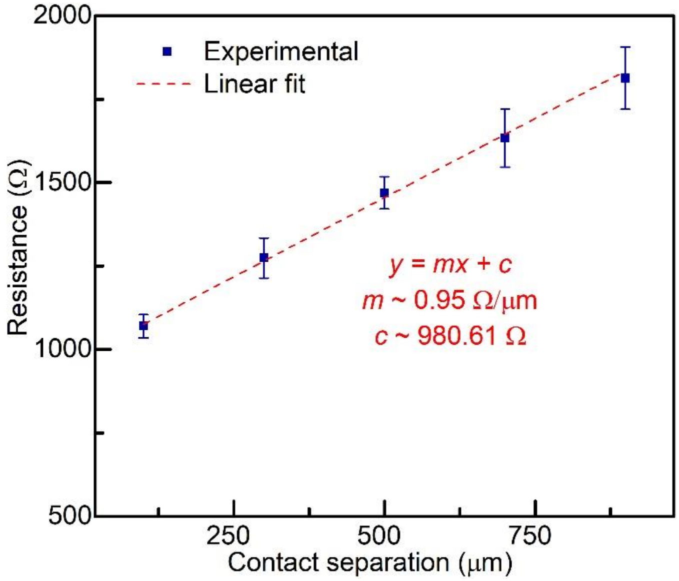

Fig. S5: Transmission line measurement (TLM) data and linear fit to obtain the contact and sheet resistance of our ion beam deposited ITO thin films. In the limit of a zero-length resistor, the residual resistance is just twice the contact resistance; which can be calculated from the intercept. Also the sheet resistance of the film can be found from the slope of the line and channel width i.e. contact width of 100 μm.

used and one probe was kept stationary on the first contact pad and the second probe was placed onto the adjacent pad. A voltage sweep from -1 V to 1 V marked the corresponding current flow data into a LabView program plotting voltage vs current and an expected linear trend. The corresponding resistance was recorded by division of the voltage and current data. This process was repeated for each measurement from -1 V to 1 V volts in 0.14 V steps followed by an averaging for this set of measurements. The second probe was then moved onto the next contact pad spanning a total distance of 300 μm away from the first. The same process of sweeping the voltage, finding and averaging the resistance was repeated. Overall, this process of moving the probe and finding the resistance was repeated several times for each length.

A linear plot of averaged resistances versus length (in nm) is presented. From this data, the contact and sheet resistance were found approximately at 490 Ω and 95 Ω/□. Such TLM methods

can suffer from fringe field component effects as the conductive ITO film was not patterned to fit only width of the contacts. Consequently, we carried out Hall effect measurements to find the mobility and carrier concentration of the as deposited films. Hall effect measurements and 4-point probe measurements sufficiently agree in the obtained resistivity of our deposited ITO films as 8.12×10^{-4} Ω-cm. The mobility was found as 24.91 cm^2/V-s.

References

[1] J. W. Cleary, E. M. Smith, K. D. Leedy, G. Grzybowski, and J. Guo, "Optical and electrical properties of ultra-thin indium tin oxide nanofilms on silicon for infrared photonics," Opt. Mater. Express **8**(5), 1231-1245 (2018).

[2] C. Zhang, N. Hong, C. Ji, W. Zhu, X. Chen, A. Agrawal, Z. Zhang, T. E. Tiwald, S. Schoeche, J. N. Hilfiker, L. J. Guo, and H. J. Lezec, "Robust extraction of hyperbolic metamaterial permittivity using total internal reflection ellipsometry," ACS Photonics **5**(6), 2234–2242 (2018).
